# A novel immunohistochemical protocol for paraffin embedded tissue sections using free-floating techniques

**DOI:** 10.3389/fnana.2023.1154568

**Published:** 2023-05-10

**Authors:** Carolina Muniz Partida, Eric Walters

**Affiliations:** Department of Biochemistry and Molecular Biology, Howard University College of Medicine, Washington, DC, United States

**Keywords:** neuroscience, neurotransmitters, immunodetection, immunofluorescence, mouse brain

## Abstract

Immunohistochemistry (IHC) is a well-established and widely used protocol used to visualize tissue architecture, protein expression and localization. Free-floating methods for IHC employ tissue sections that are cut from a cryostat or vibratome. The limitations of these tissue sections are tissue fragility, poor morphology, and the need to use sections of 20–50 μm. In addition, there is a void of information regarding the use of free floating immunohistochemical techniques on paraffin embedded tissue. To address this, we developed a free-float IHC protocol with paraffin embedded tissue (PFFP) that saves time, resources, and tissues. PFFP localized GFAP, olfactory marker protein, tyrosine hydroxylase, and Nestin expression in mouse hippocampal, olfactory bulb, striatum, and cortical tissue. Successful localization of these antigens was achieved using PFFP with and without antigen retrieval, with subsequent chromogenic DAB (3,3′-diaminobenzidine) development and immunofluorescence detection methods. The application of the PFFP in combination with methodologies of *in situ* hybridization, protein/protein interactions, laser capture dissection, and pathological diagnosis expands the versatility of paraffin embedded tissues.

## Introduction

Immunohistochemistry (IHC) is one of the most common applications used to identify tissue structure and to detect the expression of specific antigens. Preparation of tissue samples and their preservation is critical to maintain the integrity of cell morphology and architecture (Ramos-Vara, 2005; Ramos-Vara and Miller, 2014). Commonly used methods to prepare tissues for IHC are paraffin infiltration, vibratome sectioning and cryo-preservation. These methods have proven as reliable tools for detection for detailed mapping of neurotransmitters and neuronal circuits in the nervous system using IHC.

Cryo-sectioning of tissues is fast, easily prepared, and provides a good system to visualize and detect antigens within freshly cut, cryopreserved tissue (Fischer et al., 2008; Potts et al., 2020). In this regard, cryosections can be used for multiple procedures, such as immunochemistry, *in situ* enzymatic assays, hybridization and enzymatic detection. Although cryosections are physically less stable and of reduced morphological quality than paraffin tissue, they offer significant advantages to preserve epitope specificity and detection of antigen labeling (Fischer et al., 2008; Chen et al., 2010).

Paraffin processing of tissues is routinely employed because of its cost-effectiveness, preservation of detailed morphology, and efficacy for extended storage of embedded samples. This differs significantly from requirements for special handling procedures, tissue sensitivity, and limited shelf life of cryo-preserved tissue (Kokkat et al., 2013). Challenges with use of formalin-based methods of fixation, however, may render some antigens “invisible” to antibody detection when performing IHC. To compensate for this, antigen retrieval techniques such as incubation with citrate buffer, mild acids and heat treatment have been utilized to improve antigenicity (Kokkat et al., 2013; Ward, 2018). Additionally, slide mounted paraffin and cryosections may both require higher titer of antibody due to limited access of antibody to the entire section (Ravikumar et al., 2014). In this regard, free-floating tissue protocols for IHC and immunofluorescence are popular alternative strategies that researchers utilize to localize and quantify expression of proteins *in situ*. Free floating techniques involve suspension of tissue sections in antibody solution, and is now a well-established method for cryostat and vibratome tissue sections (ranging from 20 to 50 μm thick).

Recently, a paraffin-embedded tissue fragment suspension method (PETFS) for IHC analysis of pathological markers determined that batch tissue sections stored in ethanol for extended periods displayed identical detection when compared to slide-mounted formalin-fixed paraffin embedded (FFPE) tissue from the original donor (Ding et al., 2017). A limitation of the PETFS study was that tissue fragmentation accompanied its methodology. Whereas other groups reported impressive use of free-floating applications to stain brain tissues (Jiao et al., 1999), the development of defined and efficient protocols for fluorescent and chromogenic detection of expressed proteins in brain tissues is desirable. The PETFS is a useful tool for analysis of antigens and features of pathological specimens; however, the development of a robust and efficient protocol for free-floating IHC with complete, intact tissue sections is desirable. Such methodology has potential to improve antibody detection, antigenicity, and specificity.

Here, we outline steps for a novel Paraffin Free Float Protocol (PFFP) method for immunohistochemical localization of multiple proteins expressed within neurons and glial cells within the mouse brain. The importance of a PFFP can add versatility to IHC methodology by: (a) the use of relatively thin (5–10 μm) sections compared to cryostat and vibratome samples; (b) preservation of morphological integrity; (c) expression and localization of antigens in long-term storage tissues; and (d) employing both chromogenic (enzymatic) and fluorescence signal detection. The PFFP offers researchers certain advantages to easily identify and analyze proteins of interest in multiple types of tissue sections. Successful incorporation of a PFFP antigen detection, combined with other histochemical methods, has broad application within basic, clinical, and other applied arenas.

## Materials and methods

### Mouse brain tissue samples

Briefly, mice were anesthetized with sodium pentobarbital and transcardially perfused with cold PBS (30 ml) followed by 40 ml of Bouin’s fixative. Brains were post-fixed in Bouin’s overnight, followed by overnight rinsing in PBS, and subsequent rinses in graded ethanols (50, 70%) before being infiltrated in paraffin with an automated processor (Tissue-Tek VIP). Tissue sections from mouse olfactory bulbs and brain regions (cortex, striatal, midbrain and cerebellum) were obtained from aged (14 years old) and new (1 month old) paraffin blocks were sectioned at a 5, 8, 10, 15, and 20 μm for use in the PFFP protocol (see below).

### Deparaffinization, rehydration, and antigen retrieval

**FIGURE 1:**
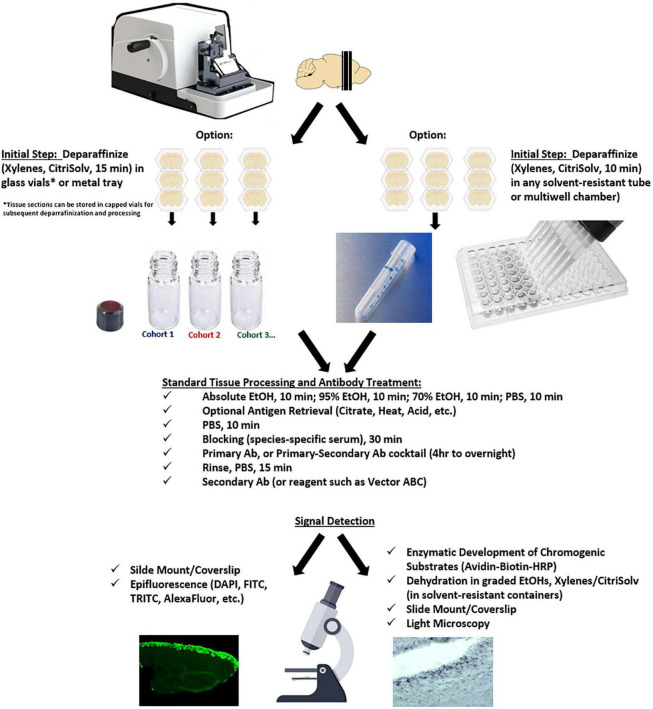
Workflow for the PFFP methodology.

A general workflow for the PFFP methodology is outlined in Figure 1, and protocols for the paraffin free floating method is provided within figure legends and in stepwise detail below. Briefly, tissue sections were placed in CitriSolv (Fisher Scientific) solution for 15 min, followed by 100, 95, 70% ethanols, and PBS (10 min each). For antigen retrieval, 0.01 M of Citrate Buffer (pH 6.0) was heated to boil and free floating sections incubated for a minimum of 10 min and returned to PBS for Cresyl Violet staining and immunohistochemistry/immunofluorescence.

### Cresyl violet stain solution (0.1%) Nissl stain

Free floating sections were placed in Cresyl Violet Stain Solution (0.1%) for 2 to 5 min, then rinsed immediately in absolute ethanol. Tissue sections were cleared for a final step in CitriSolv for 10 min and mounted on Superfrost Plus (Fisher) microscope slides with Permount (Fisher) and coverslipped.

### Immunofluorescence and peroxidase immunohistochemistry

Free floating sections were hydrated (Figure 1) and treated with and without antigen retrieval, and subsequently incubated in primary antibodies (see Table 2) targeting glial fibrillary protein (GFAP), tyrosine hydroxylase (TH), nestin, and olfactory marker protein (OMP) were used to localize expression within the olfactory bulb and regions across the CNS.

Tissue samples were treated with primary antibodies overnight at room temperature, rinsed with PBST (PBS-0.1% Tween 20), followed by 1 h incubation with species-specific secondary antibodies labeled with FITC, TRITC, and DyLight 488 fluorochromes (see Table 2) (1:80). Dual-labeling of OMP and GFAP antigens consisted of overnight incubation of primary antibody cocktail solution (anti-OMP/anti-GFAP) followed by FITC-anti-goat IgG/TRITC-anti-rabbit IgG secondary antibodies for approximately 2 h. For mounting onto glass slides, sections were “wetted” with 50% glycerol and a fine brush or tweezers was used to position sections to lay flat onto the slide and coverslipped for fluorescence microscopy.

For chromogenic detection of antigens, tissues were incubated with species-specific biotinylated secondary antibodies (40 min), followed by ABC (avidin-biotin-conjugate HRP, Vector Labs, see Tables 1, 2) for 30 min. Development of signals was monitored using 3′3′-diaminobenzidine (DAB) as a substrate, and the reaction was terminated with water. Sections were dehydrated with graded ethanols (70, 95, 100%) and cleared in CitriSolv before mounting onto glass slides.

**TABLE 1:** List of all the reagents and product information/supplier.

Reagents/materials	Product information/ supplier
CitriSolv and absolute ethanol	Fisher Scientific
Metal ice tray, 8.46 × 4.45 × 1.34 inches	Manufacturer: CHICIRIS ASIN, #B09N3SD2B9 (purchased from Amazon)
Glass vials	Fisherbrand™ 51 Expansion Glass Shell Vials with Plug Style Closures
Polypropylene round bottom tubes	Becton Dickinson, Falcon #352059
Tissue culture plates	Fisher Scientific
Normal horse serum	S-2000-20 Vector Laboratories
ABC reagent	Vector Laboratories
Vectashield Antifade mounting medium with DAPI	Vector Laboratories
SuperFrost plus microscope slides, coverslips	Fisher Scientific
Permount™	Fisher Scientific

**TABLE 2:** List of primary and secondary antibodies used in the PFFP study.

Primary antibodies	Secondary antibodies (chromogenic development)	Secondary antibodies (fluorescent detection)
DAKO monoclonal GFAP rabbit #Z0334 (1:200)	Vector Laboratories goat anti-rabbit IgG antibody (H + L), peroxidase #PI-1000-1 (1:250)	DyLight 488, goat anti-rabbit IgG, Thermo Fisher (1:80); Santa Cruz Biotechnology, mouse anti-rabbit IgG (Rhodamine) #sc-2492 (1:100)
WAKO monoclonal OMP goat #019-22291 (1:100)	N/A	Vector Laboratories, rabbit anti-goat IgG antibody (H + L) FITC; AbClonal donkey anti-goat IgG (FITC) #AS032 (1:100)
Developmental Studies Hybridoma Bank monoclonal Nestin mouse #AB_2235915 (5 μg/ml)	N/A	DAKO rabbit anti-mouse TRITC #R0270 (1:80)
ABCAM monoclonal Tyrosine Hydroxylase rabbit #ab112 (1:750)	Vector Laboratories goat anti-rabbit IgG antibody (H + L), peroxidase #PI-1000-1 (1:250)	DAKO swine anti-rabbit TRITC #R0156 (1:80) DyLight 488, goat anti-rabbit IgG, Thermo Fisher (1:80)

## Results

**FIGURE 2:**
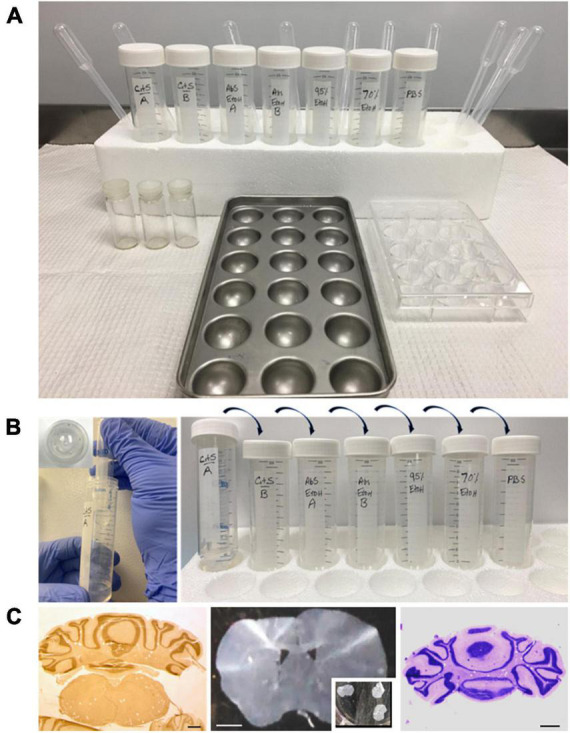
Set up for deparaffinization and hydration of brain paraffin embedded brain tissues for the PFFP protocol. **(A)** Tissue sections are placed in vials or in the metal pan and are incubated for a minimum of 10 min in CitriSolv, graded alcohols (100, 95, and 70%) and PBS to achieve hydration. Sections can be transferred to cell culture plates, or a perforated tube [**(B)**, see below], for further processing for histology or immunohistochemistry. **(B)** Illustrates a streamlined protocol for processing tissues in xylene/CitriSolv–resistant Falcon tubes. The inset shows perforation of a Falcon tube that can be sequentially processed through graded ethanols, PBS hydration. The perforated tubes can also be used for minimal amounts of antibody incubation for immunohistochemistry. **(C)** Left to right: stereo-microscope images of a coronal section of cerebellum (floating in CitriSolv solution) and striatum (fully hydrated in PBS solution, multi-well plate) of the mouse brain, which demonstrates great morphological preservation using the PFFP method. Morphological integrity of cerebellar tissue is exemplified with the PFFP method through Cresyl violet (Nissl) staining. Bar 100, 200, 100 μm.

Because many archival samples are stored in paraffin blocks, the ability to identify peptides and protein species within formalin-fixed tissues is an important aspect related to the sensitivity and specificity of an IHC protocol. Bouin’s fixative (formaldehyde, acetic acid, and picric acid) retains excellent morphological integrity, was ideal to determine efficacy of the paraffin free-floating protocol (PFFP) for aged tissues blocked and retained at room temperature in our laboratory for 14 years. Coronal sections of varying thicknesses (5, 8, 10, and 20 μm) of the brain were subjected to solutions for deparaffinization, graded ethanols, and eventual rehydration in PBS (Figure 2A). As a general rule, tissue sections were initially deparaffinized in glass vials or metal trays that were resistant to xylenes or CitriSolv reagents. Upon rehydration, tissues were routinely transferred to tissue culture plates or microtiter plates for remaining steps in the PFFP method. We also streamlined the PFFP process by employing a Falcon tube that was perforated at its base (5 holes, 26 gauge needle) to facilitate efficient incubation and transfer between reagents (Figure 2B). Five micron sections of cerebellum and midbrain striatum that were deparaffinized in bulk (*n* = 3–10 per vial) were stable during manipulation and transfer between each solution of the protocol, retained their morphological integrity during antigen retrieval techniques, and were optimal for general histological staining (Figure 2C).

**FIGURE 3:**
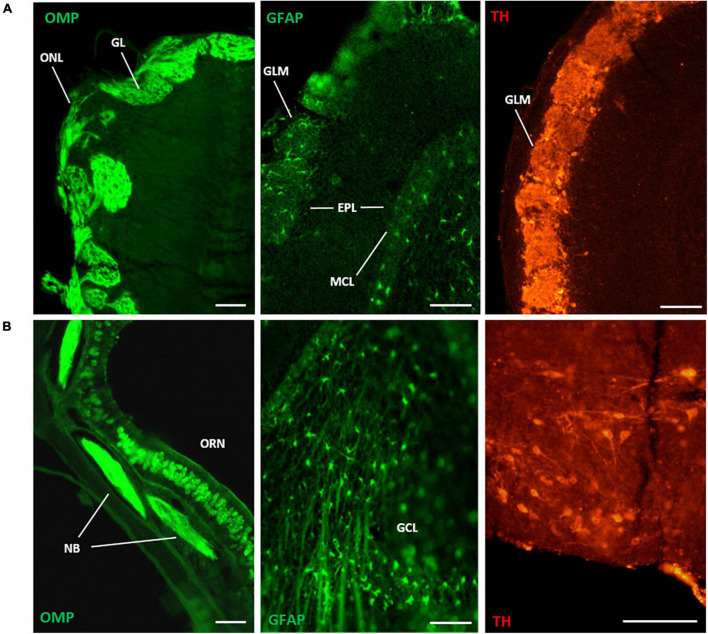
Immunofluorescence detection of olfactory bulb antigens with the PFFP method (processed in glass vials, see Figures 1, 2A). Deparaffinized coronal sections were processed with overnight incubation with polyclonal antibodies followed by species-specific fluorescent-labeled secondary antibodies. [**(A)**, left to right], coronal section of mouse olfactory bulb (5 micron thickness) localized olfactory marker protein (OMP) in axons of the olfactory nerve layer (ONL) that extend to glomerular layers (GLM) of the main olfactory bulb. Staining of glial fibrillary acidic protein (GFAP) identified characteristic astrocytes interspersed at glomeruli and the mitral cell layer (MCL) of the bulb (8 micron thickness). Similarly, tyrosine hydroxylase expression was identified in juxtaglomerular interneurons of the GLM (8 micron thickness). [**(B)**, left to right], cell type-specific expression of OMP was localized to primary olfactory neurons (ORN) in the olfactory nasal cavity (8 micron thickness), where nerve bundles (NB) coalesced in subepithelial regions. GFAP expression revealed stellate radial astrocytes with long projections throughout the inner, granule cell layer (GCL) of the bulb (8 micron thickness). Robust TH expression localized to neurons of the arcuate nucleus near the hypothalamus (10 micron thickness). Scale bars (left to right): **(A)** = 100 μm, **(B)** = 40, 100, 100 μm.

Primary antibodies across multiple species were effectively used at recommended manufacturers concentrations to optimize detection of antigens using the PFFP method (Table 2). Localization of multiple proteins within brain tissues was easily achieved with established, free-floating IHC with polyclonal primary and secondary (fluorescently labeled) antibodies. Distinct expression of olfactory bulb markers for neuronal and non-neuronal factors were identified within axons, glomeruli, and various layers of the bulb and in other areas of the CNS (Figure 3). Olfactory marker protein (OMP), which identifies mature olfactory receptor neurons within the primary neurons, was expressed within cell bodies, nerve bundles, and axon terminals in the bulb (Figures 3A, B). The astrocyte marker, glial fibrillary acidic protein (GFAP) identified numerous radial astrocytes that were localized to the glomeruli, and within the deeper layers of the olfactory bulb. Correlate tyrosine hydroxylase (TH) was expressed similarly within juxtaglomerular interneurons of the bulb, and in populations of neurons within the sub-hypothalamic region. The PFFP can also be performed in microtiter plates using different volumes (50–100 μl) of antibody, which is advantageous when using limited, costly reagents.

**FIGURE 4:**
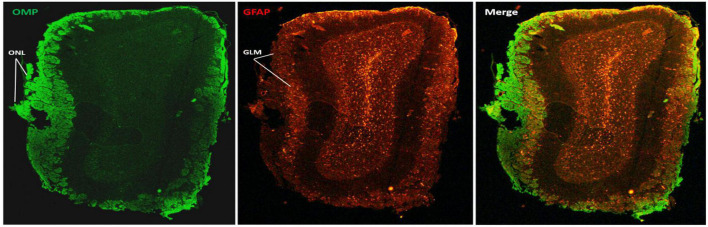
Dual immunohistochemical labeling for antigens in mouse olfactory bulb tissue using the PFFP method with perforated tubes (see Figure 2B). Deparaffinized coronal sections (5 micron thickness) with a 24 h incubation period with polyclonal antibodies followed by species-specific fluorescent-labeled secondary antibodies, demonstrating single, and or dual labeling of glial fibrillary acidic protein (GFAP) and olfactory marker protein (OMP) expression in astrocytes and neurons of the olfactory nerve layer (ONL) and glomerulus layer (GLM), respectively, and GFAP specificity to deeper layers of the olfactory bulb. The third panel illustrates a merge of the dual labeling for GFAP and OMP. Bar = 100 μm.

We explored the effect of PFFP for colocalization of (OMP) and glial fibrillary acidic protein (GFAP) in bulb tissue with dual labeling of tissues with primary antibodies, followed by 2 h incubation of secondary antibodies. Figure 4 illustrates the expression of OMP from axons of individual mature neurons that originate from the primary olfactory epithelium of the nasal cavity, culminating at the olfactory nerve layer and bulb glomeruli. It is important to note that the makeup of glomeruli consists of neuronal expression of OMP and interneuronal astroglial projections that express GFAP.

**FIGURE 5:**
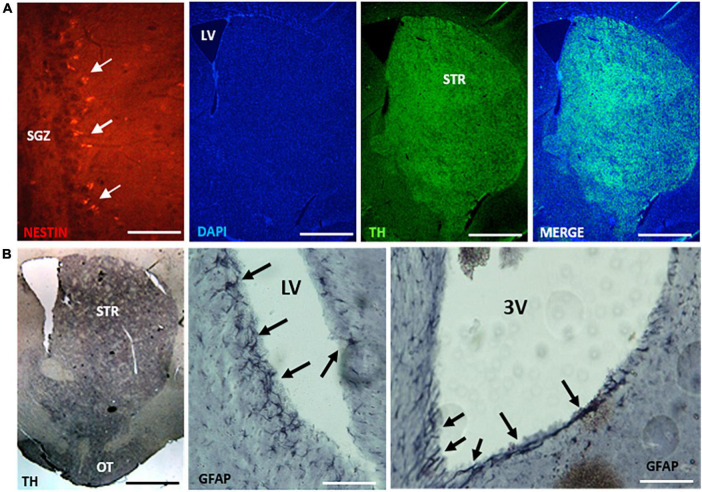
Deparaffinized coronal sections (8 μm thickness) of midbrain tissues show compatibility for immunofluorescent and chromogenic detection of antigens using the PFFP method. [**(A)**, left to right], coronal sections identified nestin-expressing cells within the subgranular zone (SGZ) of the hippocampus. PFFP method afforded staining with DAPI and TH localization in the striatum (STR). Section thickness in **(A)** 10 microns. [**(B)**, left to right], tyrosine hydroxylase (TH) and GFAP localization using peroxidase-labeled secondary antibodies and chromogenic (3, 3′-diaminobenzidine) development. Robust TH expression in the striatum correlated with similar pattern of localized expression using fluorescent detection in **(A)**. Arrows identify GFAP labeling of radial astrocytes bordering the lateral ventricle (LV) and tanycytes lining the third ventricle (3V). Section thickness in **(B)** 10 microns. Scale bars (left to right): **(A)** = 100 μm, 1, 1, 1 mm; **(B)** = 1 mm, 300, 300 μm. Coronal sections of tissues processed using multiwell plates (see Figures 1, 2A).

To ensure compatibility with both fluorescent and colorimetric detection methods of IHC, striatal tissue was subjected to the PFFP. Tyrosine hydroxylase expression in the caudate and tubercle areas of the striatum were identified using the same primary antibody concentrations for subsequent fluorescence and chromogenic (ABC-HRP) signal visualization (Figures 5A, B), and GFAP was observed in distinct radial astrocytes of the lateral ventricles adjacent to the striatum, and tanycytes that lined the third ventricular areas near the hypothalamus (Figure 5B).

## Discussion

Immunohistochemistry (IHC) is a popular technique that is regularly used to detect multiple antigen expression and localization in the peripheral and central nervous system. The use of free-floating tissue protocols has been applied across multiple areas of neuroscience research to elucidate structural and functional roles of neurotransmitters, ligand receptor interaction, protein trafficking, and organellar dynamics. The paraffin-based free floating protocol (PFFP) method for immunohistochemical detection reported herein will be beneficial for the localization of multiple antigens in brain tissues across many species.

Cresyl violet staining of tissue sections showed that PFFP ensured morphological integrity of aged samples of mouse brain and were amenable to general histological staining. Following this, we successfully identified individual expression of GFAP, olfactory marker protein (OMP), and tyrosine hydroxylase (TH) in olfactory bulb tissues, and correlate expression of GFAP, TH, and nestin within various regions of the striatum, hippocampus, and ventricles of the mouse brain. In addition, dual labeling of GFAP and OMP was easily achieved with PFFP in the olfactory bulb. Antigens were detected with standard incubation protocols, consisting of overnight incubation with primary antibody, followed by a 2 h, dual cocktail of secondary antibodies (Figure 4). The versatility of the PFFP was also applicable with standard methods of chromogenic (DAB) detection of antigens in astrocytes and striatum (GFAP and TH, respectively) that correlated with fluorescence secondary antibody labeling. In this regard, the PFFP method has enormous versatility when combined application of fluorescent and chromogenic detection is necessary to better elucidate functional relationships of factors expressed within specific cells or tissues.

Paraffin embedded tissue samples remain intact for decades, preserving morphology and cytoarchitecture. One of the limitations of archival tissue may be the degradation of proteins and the gradual loss of antigenicity, whereby various antigen retrieval techniques are applied to generally improved access of antibodies to enhance detection and specificity. Because PFFP localized various target proteins in membranous, intracellular, and nuclear areas of mouse brain on 5–10 μm tissue sections, we envision that this method will be a valuable tool to overcome limitations of IHC detection associated with slide-mounted tissues, and may offer antibody penetration that is valuable for 3D reconstruction of nervous tissues. In this regard, the application of PFFP and associated protocols offers an immense value to improve antibody specificity within tissues, for identification of previously undetectable proteins. PFFP methodology may facilitate improved localization of proteins, nucleic acid sequences, and could prove a valuable tool to medical and teaching universities, museums and archeological disciplines. Moreover, archived paraffin tissues subjected to PFFP, combined with enhanced detection strategies (Piña et al., 2022) may offer additional promise within clinical and pathological settings for accurate diagnoses (Blows et al., 2016). We note that traditional pathology related archived tissue (4% neutral buffered Formalin Fixed Paraffin Embedded, FFPE) is distinctly different than Bouin’s fixative (composed of 5% acetic acid, 9% formaldehyde, 1.5% picric acid) and such distinctions should be acknowledged pertaining to researcher and clinical terminologies. Furthermore, the incorporation of PFFP with other technologies, such as laser capture microdissection (in clinical and non-clinical settings) will allow for more precise harvesting of single or multiple cells for downstream applications related to protein expression, RT-PCR, transcriptome, and genome analyses (Matas et al., 2010; Datta et al., 2015; Roudnický et al., 2020).

Free-floating methods of IHC with cryostat and vibratome tissue sections has been an effective tool for antigen localization. By extension, the PFFP method allows researchers to employ free-floating strategies for immunodetection of proteins within formalin-fixed paraffin embedded tissues. Whereas this report of the PFFP method employed mouse brain, we foresee its broad application to studies in human brain tissues. The PFFP can prove to be a valuable and indispensable technique that better enables researchers to study the localization, distribution, and quantification of proteins in tissues or cells, with the benefit of utilizing minimal reagents for cost savings.

## Detailed general steps in the PFFP methodology

*Note: all solutions used for deparaffinization and rehydration at a ratio minimum of 200 μl per tissue section.

(1) Deparaffinize and hydrate tissue sections:

a. CitriSolv          7.5 min.
b. CitriSolv          7.5 min.
c. Absolute EtoH          10 min.
d. 95% EtoH           10 min.
e. 70% EtoH           10 min.
f. Phosphate buffered saline (PBS) 10 min.
g. (optional) antigen retrieval in 0.01 M Citrate, pH 6.0, 10 min; followed by PBS.
h. Blocking (species-specific serum) 10–30 min.

(2) Treatment with primary and secondary antibodies:

a. Incubate sections overnight with primary Ab (diluted in PBS) at room temperature.
b. Rinse in PBS, 10 min with shaking.
c. Incubate sections in fluorescent or biotinylated* secondary antibodies for 45 min.
d. Rinse in PBS, 10 min with shaking.
e. For fluorescent detection: mount tissue sections (see detailed protocol below^**^) onto SuperFrost Plus slides (glycerol: PBS, 1:1) and view on microscope.

### *For ABC detection (chromogenic development)

a. Prepare ABC (Avidin-Biotin Conjugate) 30 min in advance.
b. Incubate biotinylated tissues for 45 min.
c. Rinse in PBS, 10 min, with shaking.
d. Prepare colorimetric (DAB solution).
  20 mg of diaminobenzidine.
  48 ml of PBS (or buffer).
  240 μl of 8% Nickel Chloride (8% in H_2_O).
  16 μl of 30% Hydrogen Peroxide (added just prior to use).
e. Rinse in PBS, 10 min, with shaking.
f. Dehydrate tissues in graded EtOH (70, 95, 100%), 10 min each.
g. CitriSolv, 7.5 min.
h. CitriSolv, 7.5 min.
i. Mount tissue sections (Permount) onto SuperFrost Plus slides and view on microscope.

### ^**^Mounting free-floating sections

(1) Wet the slide with 50% glycerol solution before placing the section onto the slide.

(2) Gently suction one or more sections and deposit tissue sections onto a microscope slide; gently “pulse” the glycerol solution to encourage unfolding of tissue. Smooth gentle motions will ensure success.

(3) Gradually withdraw and elevate the slide from the mounting solution. Carefully pipette glycerol off the slide while adjusting the section to its proper position.

(4) Place one drop of Vectashield/DAPI mounting solution on the slide; coverslip.

## Data availability statement

The original contributions presented in this study are included in the article/supplementary material, further inquiries can be directed to the corresponding author.

## Author contributions

CMP participated in the concept, design, experimentation, and primary authorship. EW participated in the concept, design, experimentation, authorship, and supervision of the work. Both authors contributed to the article and approved the submitted version.
